# The Prognostic Importance of TAPSE in Early and in Stable Cardiovascular Diseases

**DOI:** 10.3390/jcdd7010004

**Published:** 2020-01-15

**Authors:** Paolo Giovanardi, Enrico Tincani, Marco Maioli, Stefano Tondi

**Affiliations:** 1 Cardiology Service, Department of Primary Care, Azienda USL, Via del Pozzo N 71, 41100 Modena, Italy; 2 Cardiology Division, Ospedale S. Agostino–Estense, Azienda Ospedaliero-Universitaria, Via Giardini 1355, 41126 Baggiovara, Modena, Italy; p.giovanardi@gmail.com; 3 Internal Medicine Division, Ospedale S. Agostino–Estense, Azienda Ospedaliero-Universitaria, 41100 Modena, Italy; tincani.enrico@aou.mo.it; 4 Department of Physics, Informatics, and Mathematics, University of Modena and Reggio Emilia, 41100 Modena, Italy; marco.maioli@unimore.it

**Keywords:** right ventricular function, TAPSE, MACES, myocardial infarction, unstable angina, heart failure, stroke

## Abstract

The identification of predictors of major cardiovascular events (MACES) represents a big challenge, especially in early and stable cardiovascular diseases. This prospective study comparatively evaluated the prognostic importance of left ventricular (LV) and right ventricular (RV) systolic and diastolic function, pulmonary artery pressure (PAP) and pulmonary vascular resistance (PVR) in a stable patient’s cohort with cardiovascular risk factors. The LV ejection fraction, mitral annular plane systolic excursion (MAPSE), tricuspid annular plane systolic excursion (TAPSE), functional mitral regurgitation (FMR), doppler tissue imaging of mitral and tricuspid annulus with systolic and diastolic peaks estimation, tricuspid regurgitation velocity (TRV), pulmonary velocity outflow time integral (PVTI), mean pulmonary artery pressure (MPAP) and PVR were estimated at enrollment. During the follow-up, MACES and all-cause mortality were recorded. 369 subjects with or without previous MACES were enrolled. Bivariate analysis revealed LVEF, TAPSE, MPAP, TRV, PVR, LV diastolic function, and FMR were associated with the endpoints. When computing the influence of covariates to the primary endpoint (all-cause mortality and MACES) through Cox analysis, only LV diastolic function and TAPSE entered the final model; for the secondary endpoint (MACES) only TAPSE entered. TAPSE was able to predict MACES and all-cause mortality in early and stable cardiovascular diseases. The use of TAPSE should be implemented.

## 1. Introduction

Many clinical variables and ultrasound parameters play a prognostic role in advanced or unstable cardiovascular diseases [1]. The identification of predictors of major cardiovascular events (MACES) in early and stable cardiovascular diseases is even more interesting [2]. Only a few commonly used echocardiographic parameter expression of left ventricular (LV) systolic and diastolic function, functional mitral regurgitation (FMR), and right ventricular (RV) function play a prognostic role in overall population and in stable patients with previous MACES [3,4,5,6]. Pieces of evidence are increasing in this field and new echocardiographic parameters, such as global longitudinal strain, seem to have an additional value [7].

Until now, to our knowledge, in stable patients with cardiovascular risk factors a comparative study has not been conducted yet. This prospective, observational cohort study was designed to ascertain the prognostic value of old and new ultrasound parameters of LV and RV systolic and diastolic function (principally derived from mitral and tricuspid annular motion), FMR, pulmonary artery pressure (PAP) and pulmonary vascular resistance (PVR) in stable patients with cardiovascular risk factors with or without previous MACES.

## 2. Methods

This study was performed in accordance with the Ethical Standards of the 1975 Helsinki Declaration revised in 2013. The study was approved by the Ethics Committee of Modena (protocol number 238/2010, date of approval 13 April 2011) and written informed consent was obtained from participants before the enrollment.

Patients referred to our Echolab, from October 2011 through August 2014, were eligible. The data of patients about medical history and cardiovascular risk factors were collected. Subjects with at least one of the major cardiovascular risk factors or with previous MACES (myocardial infarction and unstable angina, overt heart failure decompensation and stroke) that were stable during the last 12 months, were eligible. Patients with MACES during the last 12 months were excluded. Patients suffering from severe valvular diseases, chronic obstructive pulmonary disease, severe pulmonary hypertension, congenital heart disease, atrial fibrillation, with cardiac stimulators and with bad acoustic windows were also excluded.

According to the recommendation of the American Society of Echocardiography/European Association of Cardiovascular Imaging (ASE/EACVI), at enrollment a complete echocardiographic study was performed including M-mode, two-dimensional, pulsed and continuous Doppler spectral recording, as well as Doppler tissue imaging (DTI) evaluation of the mitral and tricuspid lateral annulus [8,9]. Two-dimensional images were obtained in parasternal long and short-axis views, in the apical 4- and 2-chambers view and in the subcostal view. Chambers size and wall thickness were measured [10,11].

Two echocardiographic systems (Sequoia 512–Acuson Siemens, Mountain View, USA; Vivid E9–GE Healthcare, Norwalk, USA) were used. For each patient the following parameters were estimated: LV ejection fraction (EF), mitral and tricuspid annular plane systolic excursion (MAPSE, TAPSE), FMR, pulmonary valve outflow time-velocity integral (PVTI), tricuspid regurgitation velocity (TRV), mean pulmonary artery pressure (MPAP) and PVR [12]. Mitral and tricuspid pulsed Doppler flow and DTI of the mitral and tricuspid annulus were evaluated. LV and RV systolic and diastolic peaks were estimated and the multiparameter evaluation of LV and RV diastolic function was then assessed.

LV EF was measured through the biplane method of discs; the modified Simpson’s rule was obtained from the apical 4- and 2-chamber view by determining the end-diastolic and the end-systolic volume. Reduced LV EF was defined as <55% [13].

MAPSE represents the systolic movement of the base of the LV free wall; it was measured in the apical 4-chambers view, determining with the M-mode guide the maximal excursion of the LV free wall at the junction with the mitral valve plane from the lowest position to the systolic peak. Reduced MAPSE was defined as <1.5 cm [14].

TAPSE represents the longitudinal function of the RV by determining, with the M-mode guide, the maximal systolic excursion of the RV free wall at the junction with the tricuspid valve plane in the apical 4-chambers view. As with other regional methods, it assumes that the displacement of the basal segment is representative of the entire RV function. Reduced TAPSE was defined as <1.7 cm [15].

FMR is a dynamic condition whose severity varies depending on loading conditions. FMR was assessed at rest through vena contracta width in the parasternal long-axis view, effective regurgitant orifice area, left atrial size and regurgitant volume. According to all these parameters, FMR was then classified into four classes: absent, mild, moderate and severe [16].

PVTI was measured placing a pulsed wave sample volume in the right ventricular outflow tract at the level of the pulmonic valve in the parasternal short-axis view.

MPAP was obtained, in the absence of pulmonary outflow tract obstruction and/or pulmonary valve stenosis, when pulmonary regurgitation was performed by applying the simplified Bernoulli equation from the early diastolic peak of pulmonary valve regurgitation [17] or was derived from pulmonary artery systolic pressure [18]. Increased MPAP was defined as >25 mmHg.

PVR was calculated from the peak of the tricuspid regurgitation velocity and PVTI placing a pulsed wave sample volume in the right ventricular outflow tract at the level of the pulmonic valve in the parasternal short-axis view [12]. Increased PVR was defined as >3 Woods Units.

LVSyP velocity was measured by determining the DTI systolic peak with the sample volume placed at the junction of the LV free wall with the mitral valve plane. Reduced LVSyP was defined as <9 cm s^−1^ [19].

RVSyP and RVPrP velocities were measured by determining DTI presystolic and systolic peaks with the sample volume placed at the junction of the RV free wall with the tricuspid valve plane. Reduced RVSyP was defined as <10 cm s^−1^ [20]; RVPrP is usually greater than RVSyP but a reference value has not been defined yet [21].

The evaluation of LV diastolic function was assessed studying the mitral inflow and DTI of the mitral lateral annulus. E and A peaks, their ratio, the E deceleration time, *e*’ and *a*’ peaks, their ratio and the E/*e*’ ratio were calculated. LV diastolic filling patterns were classified by the combined quantitative analysis of these parameters into four classes: normal, impaired relaxation, pseudonormal and restrictive [9]. Impaired LV relaxation led to low E and *e*’ velocity, high A and *a*’ velocity, decreased E/A ratio and increased E deceleration time. The pseudonormal filling pattern cannot be recognized only with the evaluation of the mitral inflow pattern but needed an additional assessment, such as the Valsalva manoeuvre, *e*’ evaluation and E/*e*’ ratio estimation. The restrictive pattern showed a higher E wave, greater E/A ratio and E/*e*’ ratio [22].

RV diastolic function was assessed studying the tricuspid inflow and DTI of the tricuspid lateral annulus. From the apical 4-chamber view, the Doppler beam should be aligned parallel to the RV inflow; alignment could be facilitated by displacing the transducer medially toward the lower parasternal region. The parameters used to assess RV diastolic function were the same as those used to assess the LV diastolic function. E and A peaks, their ratio, *e’* and *a’* peaks, their ratio and the E/*e*’ ratio were estimated. RV diastolic function was then classified according to these parameters into three classes: normal, impaired relaxation and restrictive [11].

All the echocardiographic parameters were measured at end-expiration during quiet breathing, and three measurements on consecutive heart cycles were averaged. Special care was given to obtain an ultrasound beam parallel to the direction of the annular motion and the transvalvular flows, and also to optimize the focus, gain, and compression setting (to obtain the most accurate endocardium visualization). If necessary, echocardiographic parameters were calculated from multiple views. Intra- and inter-observer variability was calculated.

The enrolled patients were followed-up with every 6 months through clinical-electrocardiographic evaluation or through hospital databases consultation. During the follow-up period MACES (myocardial infarction and unstable angina, overt heart failure decompensation and stroke) and all-cause mortality were recorded.

## 3. Statistical Analysis

Continuous variables are displayed as means ± standard deviation, while categorical data are displayed as frequencies. A two-tailed *p* value ≤ 0.05 was considered statistically significant, with 95% confidence interval.

Bivariate analysis was used to find which ultrasound parameters were associated with all-cause mortality and MACES (primary composite endpoint) and with MACES (secondary endpoint). An independent-sample t-test was used for continuous variables and a Chi-squared test for categorical variables. Cox regression analysis was used to find a model predictive for the endpoints; only the meaningful parameters found at bivariate analysis were entered into the Cox model, stratifying according to the presence of previous MACES. The sample size was calculated. SPSS/PC release 2013 (IBM Corp., Armonk, NY, USA) was used.

## 4. Results

A total of 1667 consecutive patients referred to our Echolab were assessed for study eligibility; of these 369 were enrolled (mean follow-up 1178 ± 391 days). Table 1 shows the clinical features, the prevalence of cardiovascular risk factors, drug treatments and exclusion criteria. Table 2 shows the mean values and frequencies of the estimated ultrasound parameters in the entire cohort and in patients with and without previous MACES. 

**Figure 1:**
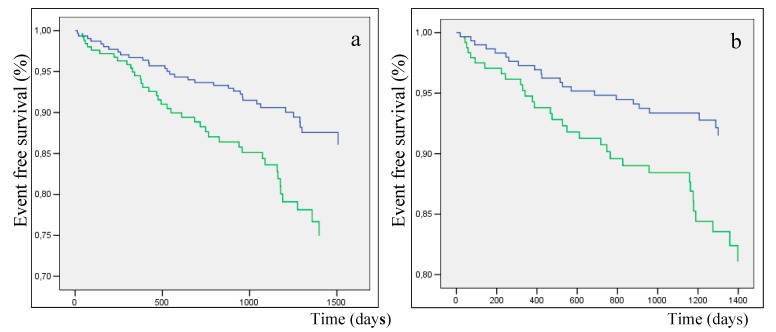
Plots of the Cox regression model. Panel (**a**) shows event-free survival for primary endpoint in patients with (lower line) or without (upper line) previous MACES according to variables entered in the Cox model (LV diastolic function and TAPSE). Panel (**b**) shows event-free survival for secondary endpoint in patients with (lower line) or without (upper line) previous MACES according to variables entered in the Cox model (TAPSE).

During the follow-up period 55 MACES were recorded (20 myocardial infarctions–unstable anginas, 27 heart failure decompensations and 8 strokes) and 29 patients died (all-cause mortality). Bivariate analysis revealed the following parameters were related to all-cause mortality and MACES: LVEF, TAPSE, MPAP, TRV, PVR, LV diastolic function, and FMR (Table 3). When computing the influence of covariates on the primary composite endpoint (all-cause mortality and MACES) through Cox analysis, LV diastolic function and TAPSE entered the final model (Table 4). When computing the influence of covariates on the secondary endpoint (MACES) through Cox analysis, only TAPSE entered the final model. Both the primary and the secondary endpoint were more frequent in patients with previous MACES (Figure 1a,b). Sample size turned out to be appropriate.

## 5. Discussion

Cardiovascular risk factors have an early influence on the heart, vessels and lungs [23]. Previous studies demonstrated the prognostic importance of the left heart also in early cardiovascular diseases [3] but we do not know so much about the right heart, for a long time considered a useless bystander.

Concerning the LV, RV has peculiar characteristics such as a larger volume, a greater longitudinal contraction and a smaller mass. RV has a greater dependence on preload (determined by RV stroke volume, tricuspid and pulmonary regurgitation) and on afterload (determined by the forces that oppose RV output and a reflection of PAP and PVR) [24]. PAP is determined by cardiac output, properties of the vasculature (resistance, capacitance and impedance) and atrial filling pressure. The assessment of RV afterload highlights the fascinating role played by PVR, closely influenced by pressure changes [25]. Moreover, LV and RV function are influenced by ventricular interdependence [26]. These interrelations are not well known, but we can hypothesize a clinical role for RV also in the early stages of cardiovascular diseases.

This study simultaneously analyzed old and new echocardiographic indexes of LV and RV function derived from annular motion, PAP and PVR and revealed the lack of importance of most of the considered parameters. In early and stable cardiovascular diseases echocardiography did not provide a powerful prognostic role except for LV diastolic function, for the primary endpoint, and TAPSE, for both the primary and the secondary endpoints. Most of the studied parameters and TAPSE were within the normal range but TAPSE turned out to be a more powerful predictor of outcome than LV function, FMR and PAP, known predictors of MACES and mortality in advanced cardiovascular diseases [4,5].

The clinical importance of RV function in the early stages has not been completely identified. We previously reported in a small cohort of stable outpatients with a poorer echocardiographic evaluation that TAPSE—within the normal range—was able to predict MACES [27]. Moreover, PAP, related to RV function, demonstrated to be a powerful predictor of mortality in the general population of the Olmsted Country [28].

In the overall population of the Copenhagen City Heart Study with cardiovascular risk factors, Modin and colleagues have recently shown that TAPSE was an independent predictor of cardiovascular death as an expression of LV diastolic dysfunction [29]. This study, in a smaller cohort with a more complete echocardiographic evaluation, confirms Modin et al.’s observation about TAPSE and LV diastolic function. They are probably linked to each other, and early expression of pressure and volume overload.

TAPSE is easy to measure, reproducible and has unique characteristics which derive from forces that contribute to RV preload and afterload [15]. Similarly to other regional methods, it assumes that the displacement of the free wall basal segment represents the entire RV function, an assumption that is less valid when there are regional wall motion abnormalities. However, TAPSE has many validations and many studies support its utility [11].

TAPSE represents the great longitudinal contraction of the RV and seems to early perceive vascular stiffness and increased preload and afterload. RV function indexes derived from DTI are not useful in the early stages since they are less load-dependent, while the left heart is a powerful structure whose function is usually preserved, especially if measured with standard techniques [7].

## 6. Limits

The cohort was heterogeneous: patients in primary prevention with cardiovascular risk factors and stable patients in secondary prevention were enrolled. Moreover, cardiovascular risk factors decline with different mechanisms of cardiac, vascular and lung function. The echocardiographic evaluation was made only with standard techniques, especially derived from annular motion.

## 7. Conclusions

This study confirms that TAPSE has a pivotal role between the LV and RV function. Larger studies are required but pieces of evidence are growing: the simple use of TAPSE in early and stable cardiovascular diseases should be implemented and, for this purpose, TAPSE limits probably should be reconsidered.

## Figures and Tables

**Table 1:** Clinical characteristics of the enrolled patients.

Clinical Characteristics:		Prevalence of Cardiovascular Risk Factors:		Cardiovascular Diseases:	
Patients who completed the follow-up §	369	Diabetes §	76 (20.6%)	Prior stroke §	34 (9.2%)
Caucasian §	367 (99.4%)	Arterial hypertension §	284 (77%)	Prior myocardial infarction/unstable angina §	53 (14.4%)
Men §	198 (53.7%)	Dyslipidemia §	254 (68.8%)	Prior overt heart failure §	26 (7%)
Mean age at enrollment date (years) *	68.1 ± 13.7	Current tobacco use §	92 (24.9%)	Patients with previous MACES °	113
Mean follow-up (days) *	1178 ± 391	**Medications for Cardiovascular Conditions:**		Patients without previous MACES	256
Mean systolic blood pressure (mmHg) *	132 ± 7	Antiplatelet agents §	196 (53.1%)	**Exclusion Criteria:**	
Mean diastolic blood pressure (mmHg) *	83 ± 4.2	ACE-I ^§	234 (63.4%)	Recent heart failure decompensation §	490
Absence of LV # hypertrophy §	175 (47.5%)	ARBs ^^§	67 (18.2%)	Atrial fibrillation §	255
Mild LV hypertrophy §	141 (38.2%)	Beta blockers §	133 (36%)	Recent acute myocardial infarction §	328
Moderate LV hypertrophy §	47 (12.7%)	Calcium channel blockers §	199 (53.9%)	COPD \, severe pulmonary hypertension and severe valvular diseases §	140
Severe LV hypertrophy §	6 (1.6%)	Lipid lowering drugs §	303 (82.1%)	Congenital heart diseases §	33
Body mass index (BMI) *	23.2 ± 4.2	Hypoglycemic agents §	70 (19%)	Bad acoustic window §	52

§, Number of patients; %, Percent of patients; *, Mean ± standard deviation; °, Major cardiovascular events; ^, Angiotensin converting enzyme-inhibitors; #, Left ventricular; ^^, Angiotensin II receptors blockers; \, Chronic obstructive pulmonary disease.

**Table 2:** Mean values and frequencies of the estimated ultrasound parameters.

LV+ Echocardiographic Parameters:				RV # Echocardiographic Parameters:			
	Entire Cohort	Previous MACES ”	No Previous MACES		Entire Cohort	Previous MACES	No Previous MACES
LV ejection fraction (%) *	52.7 ± 5.5	49.9 ± 7.5	54 ± 3.8	TAPSE ^ (cm) *	2.5 ± 0.4	2.4 ± 0.5	2.5 ± 0.4
MAPSE ## (cm) *	1.5 ± 0.8	1.4 ± 0.3	1.5 ± 1	DTI ^^ RV Presystolic Peak (cm s^−1^) *	18.4 ± 8	17.6 ± 8.2	18.8 ± 7.9
DTI LV Systolic Peak (cm s^−1^) *	11 ± 4.4	10 ± 4.2	11.4 ± 4.5	DTI RV Systolic Peak (cm s^−1^) *	16.4 ± 5	15.7 ± 5	16.8 ± 5.1
**Functional Mitral Regurgitation:**				PVTI ° (cm) *	21.4 ± 4.4	20.7 ± 4.4	21.7 ± 4.4
absent §	97 (26.3%)	16 (4.3%)	81 (22%)	TRV °° (m s^−1^) *	2.4 ± 0.5	2.4 ± 0.5	2.4 ± 0.5
mild §	222 (60.2%)	72 (19.5%)	150 (40.7%)	MPAP | (mmHg) *	21.2 ± 5.6	21.9 ± 6.1	20.9 ± 5.3
moderate §	50 (13.5%)	25 (6.8%)	25 (6.8%)	PVR -- (Woods Unit) *	1.32 ± 0.3	1.4 ± 0.4	1.3 ± 0.3
**LV diastolic function:**				**RV diastolic function:**			
normal §	51 (13.8%)	6 (1.6%)	45 (12.2%)	normal §	71 (19.3%)	18 (4.9%)	53 (14.4%)
impaired relaxation §	280 (75.9%)	85 (23.1%)	195 (52.8%)	impaired relaxation §	295 (79.9%)	94 (25.5%)	201 (54.5%)
pseudonormal §	34 (9.2%)	18 (4.9%)	16 (4.3%)	restrictive §	3 (0.8%)	1 (0.2%)	2 (0.5%)
restrictive §	4 (1.1%)	4 (1.1%)	--				

+, Left ventricular; #, Right ventricular; “, Major cardiovascular events; *, Mean ± standard deviation; ^, Tricuspid annular plane systolic excursion; ##, Mitral annular plane systolic excursion; ^^, Doppler tissue imaging; °, Pulmonary velocity outflow time integral; §, Number of patients; %, Percent of patients; °°, Tricuspid regurgitation velocity; |, Mean pulmonary artery pressure, --, Pulmonary vascular resistances.

**Table 3:** Results of bivariate analysis.

	MACES# or Death at Follow-Up	No MACES or Death at Follow-Up	*p* Value
LV+ ejection fraction (%) *	49.9 ± 7.3	53.5 ± 4.8	<0.001
MAPSE ” (cm) *	1.37 ± 0.3	1.52 ± 0.9	0.195
DTI ^^ LV systolic peak (cm s^−1^) *	10.2 ± 4.4	11.2 ± 4.6	0.064
**Functional mitral regurgitation:**			<0.001
absent §	10 (2.7%)	87 (23.6%)	
mild §	43 (11.8%)	179 (48.6%)	
moderate §	22 (6%)	28 (7.3%)	
**LV diastolic function:**			<0.001
normal §	1 (0.3%)	50 (13.6%)	
impaired relaxation §	56 (15.2%)	224 (60.7%)	
pseudonormal §	16 (4.3%)	18 (4.9%)	
restrictive §	2 (0.5%)	2 (0.5%)	
TAPSE ^ (cm) *	2.3 ± 0.5	2.5 ± 0.4	0.001
DTI RV ## presystolic peak (cm s^−1^) *	17.5 ± 7	18.7 ± 8.2	0.24
DTI RV systolic peak (cm s^−1^) *	15.5 ± 4.3	16.7 ± 5.3	0.064
PVTI ° (cm) *	21.1 ± 4.4	21.5 ± 4.4	0.498
TRV °° (m s^−1^) *	2.5 ± 0.5	2.3 ± 0.5	0.001
MPAP | (mmHg) *	23 ± 6	20.7 ± 5.4	0.001
PVR -- (woods unit) *	1.4 ± 0.4	1.3 ± 0.3	0.001
**RV diastolic function:**			0.258
normal §	13 (3.5%)	60 (16.3%)	
impaired relaxation §	60 (16.2%)	233 (63.1%)	
restrictive §	2 (0.6%)	1 (0.3%)	

#, Major cardiovascular events; +, Left ventricular; *, Mean ± standard deviation; “, Mitral annular plane systolic excursion; ^^, Doppler tissue imaging; §, Number of patients; %, percent of patients; ^, Tricuspid annular plane systolic excursion; ##, Right ventricular; °, Pulmonary velocity outflow time integral; °°, Tricuspid regurgitation velocity; |, Mean pulmonary artery pressure; --, Pulmonary vascular resistances.

**Table 4:** Cox regression analysis results for the significative parameters.

**Cox Regression Analysis for Primary Composite Endpoint (All-Causes Death and MACES #)**					
	**Hazard Ratio**	**95% Confidence Interval**			***p* Value**
		**Lower**		**Upper**	
LV + diastolic function	17.07	2.179–133.985			0.007
TAPSE ^ (cm)	0.555	0.325–0.95			0.032
**Cox Regression Analysis for Secondary Endpoint (MACES)**					
	**Hazard Ratio**	**95% Confidence Interval**			***p* Value**
		**Lower**	**Upper**		
TAPSE (cm)	0.493	0.261–0.931			0.029

#, Major cardiovascular events; +, Left ventricular; ^, Tricuspid annular plane systolic excursion.

## Data Availability

Database and statistical analysis are available.

## Back Matter

The first section of this research has been presented as poster during the ESC Congress 2018 (25–29 August, Munich).
